# A review on automatic analysis techniques for color fundus photographs

**DOI:** 10.1016/j.csbj.2016.10.001

**Published:** 2016-10-06

**Authors:** Renátó Besenczi, János Tóth, András Hajdu

**Affiliations:** Faculty of Informatics, University of Debrecen 4002 Debrecen PO Box 400, Hungary

**Keywords:** Biomedical imaging, Retinal diseases, Fundus image analysis, Clinical decision support, ACC, accuracy, AMD, age-related macular degeneration, AUC, area under the receiver operator characteristics curve, DR, diabetic retinopathy, FN, false negative, FOV, field-of-view, FP, false positive, FPI, false positive per image, kNN, k-nearest neighbor, MA, microaneurysm, NA, not available, OC, optic cup, OD, optic disc, PPV, positive predictive value (precision), ROC, Retinopathy Online Challenge, RS, Retinopathy Online Challenge score, SCC, Spearman's rank correlation coefficient, SE, sensitivity, SP, specificity, TN, true negative, TP, true positive

## Abstract

In this paper, we give a review on automatic image processing tools to recognize diseases causing specific distortions in the human retina. After a brief summary of the biology of the retina, we give an overview of the types of lesions that may appear as biomarkers of both eye and non-eye diseases. We present several state-of-the-art procedures to extract the anatomic components and lesions in color fundus photographs and decision support methods to help clinical diagnosis. We list publicly available databases and appropriate measurement techniques to compare quantitatively the performance of these approaches. Furthermore, we discuss on how the performance of image processing-based systems can be improved by fusing the output of individual detector algorithms. Retinal image analysis using mobile phones is also addressed as an expected future trend in this field.

## 1 Introduction

**Fig. 1:**
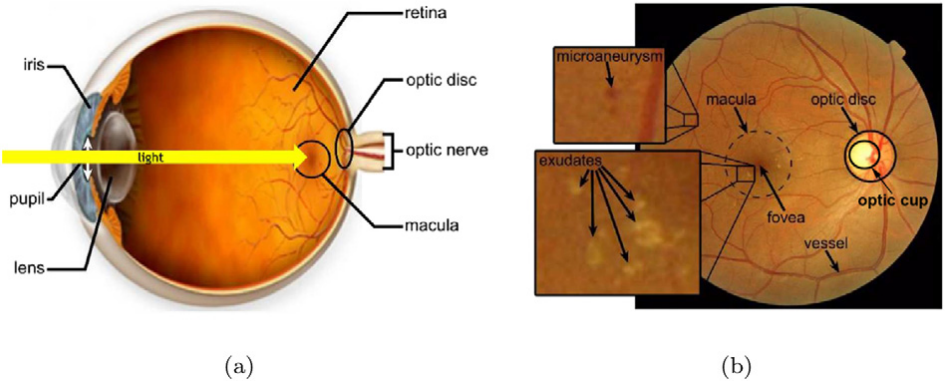
Basic concepts of retinal image analysis; (a) the structure of the human eye and the location of the retina, (b) sample fundus image with the main anatomic parts and some lesions.

The retina (fundus) [1] has a very specific diagnostic role regarding human health. The eye is a window into the body responsible for sensing information in the visible light domain, thus, it is also suitable to make clinical diagnoses in a non-invasive manner. The retina is a spherical anatomic structure on the inner side of the back of the eye as shown in Fig. 1 (a). It can be subdivided into ten layers supporting the extraction of visual information by photoreceptor cells: the rods and the cones. As any other tissue, the retina also has blood support through the vascular system, which is clearly visible from outside with an ophthalmoscope during clinical examinations. At the center of the retina a darker, round spot, the macula resides, whose center is known as the fovea, which is responsible for sharp vision. The optic disc – including the optic cup – is a bright oval patch, where the optic nerve fibers leave the eye and where the major arteries and veins enter and exit. The special structure of the retina limits the possible appearances of distortions caused by different diseases. Namely, the most common lesions appear as patches of blood or fat in retinal images. Diseases affecting the blood vessel system cause similar vascular distortions here than in any other part of the body, but are easier and better seen if examined by an experienced professional.

From diagnostic point of view, retinal image analysis is a natural approach to deal with eye diseases. However, it is getting more and more important nowadays, since the types and quantities of different lesions can be associated with several non-eye diseases, as well. In automatic image analysis, the fovea, macula, optic disc, optic cup, and blood vessels are the most essential anatomic landmarks to extract (see Fig. 1 (b)). Besides them, the recognition of specific lesions is also critical to deduce to the presence of diseases they are specific to.

In this work, we focus on automatic color fundus photograph analysis techniques to support clinical diagnoses. Accordingly, the rest of the paper is organized as follows. We highlight the major eye and non-eye diseases having symptoms in the retina in Section 2. In Section 3, we provide an overview of image acquisition techniques and summarize the most important automatic image processing approaches. These tools include image enhancement and segmentation methods to extract anatomic components and lesions. We exhibit both supervised and non-supervised techniques here. Moreover, we discuss on how the aggregation of the findings of different algorithms by fusion-based methods may improve diagnostic performance. For the quantitative, objective comparability of different approaches, we also present several publicly available datasets and the commonly applied performance measures. We discuss on possible future trends including retinal image analysis on mobile platform in Section 4. Finally, in Section 5, we draw some conclusions to provide a more comprehensive comparison of the available approaches and to give suggestions on possible improvements regarding both detection accuracy and efficient computing.

## 2 Clinical background of color fundus photograph analysis

The retina is the only site to observe vessel-related and other specific lesions *in vivo* and recent studies showed that these abnormalities are predictive to several major diseases listed next.

### 2.1 Diabetes

**Fig. 2:**
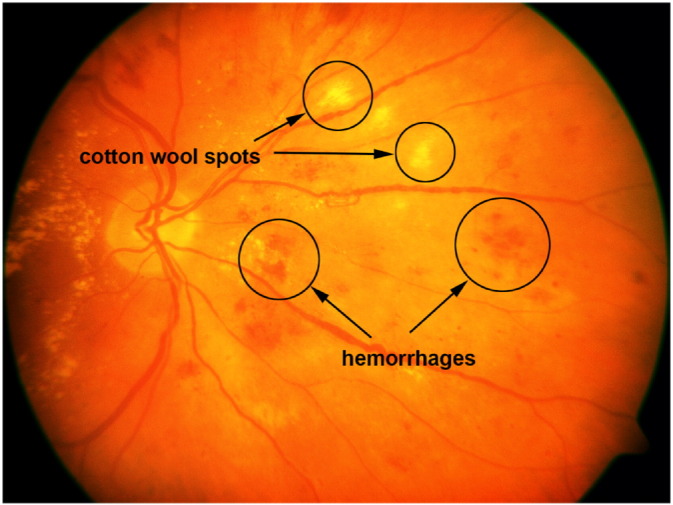
A sample retinal image with cotton wool spots and hemorrhages.

In 2015, 415 million adults suffered from diabetes mellitus [2]. This number is growing, and by 2040, it is expected to reach 642 million. Long-time diabetes affects the blood vessels also in the eyes, causing diabetic retinopathy (DR). In the case of DR, the blood vessels supplying the retina may become thick and weak, causing leaks called hemorrhages (see Fig. 2). These leaking vessels lead to swelling and edema, causing eyesight deterioration. The fluid exudates in the retina can be observed as small yellowish spots (see Fig. 1 (b)). The earliest signs of diabetes are microaneurysms (MAs, see Fig. 1 (b)), which are focal dilations of the capillaries and appear as small darkish spots. The identification of exudates, hemorrhages, and MAs are important for the early prevention of DR-caused blindness.

### 2.2 Cardiovascular diseases

#### 2.2.1 Hypertension

Wong et al. [3] summarized the major effects of systemic hypertension in the retina. Hypertensive retinopathy may cause blot- or flame-shaped hemorrhages, hard exudates, micro- or macroaneurysms, and cotton wool spots, which occur due to the occlusion of arteriole and appear as fluffy yellow-white lesions (see Fig. 2). Ikram et al. [4] pointed out that the risk of hypertension was increased with general arteriolar narrowing in the retina, mainly in the elderly population. There is a connection also between the arteriolar-to-venular diameter ratio and higher blood pressure, but with lower influence than the arteriolar narrowing. Cheung et al. [5] concluded that retinal arteriolar tortuosity was connected with higher systemic blood pressure and body mass index, while venular tortuosity was associated with lower high-density lipoprotein cholesterol level, as well.

#### 2.2.2 Coronary heart disease

Coronary heart disease is the leading cause of death worldwide. Recent studies (e.g., [6]) showed that there is a correlation between coronary heart disease and coronary microvascular dysfunction. Liew et al. [7] collected the main symptoms of microvascular dysfunctions like focal arteriolar narrowing, arteriovenous nicking and venular dilation. McClintic et al. [8] reviewed the recent findings regarding the connection between coronary heart disease and retinal microvascular dysfunction. Liew et al. [9] examined retinal vessels with fractal analysis in order to detect whether it had any connection to coronary heart disease. Their observations suggest that non-optimal microvascular branching may cause the disease. Vessel abnormalities can be characterized by geometric measures that will be discussed in Section 3.3.3.

### 2.3 Stroke

Since the cerebral and retinal vasculature share similar physiologic and anatomic characteristics, reasonable research efforts have been made in the recent years to reveal the connection between cerebral stroke and retinal vasculature. Baker et al. [10] concluded that signs of hypertensive retinopathy were associated with different types of stroke. Cheung et al. [11] showed that increased retinal microvascular complexity was associated with lacunar stroke and alterations of retinal vasculature may cause microangiopathic events in the brain. Patton et al. [12] summarized the recent advancements in the possibilities of examining the retina to search for cerebrovascular diseases.

## 3 Analysis of color fundus photographs

### 3.1 Color fundus photography

Fundus photography is a cost-effective and simple technique for trained medical professionals. It has the advantage that an image can be examined at another location or time by specialists and provides photo documentation for future reference.

Panwar et al. [13] recently collected the state-of-the-art technologies for fundus photography. Currently available fundus cameras can be classified into five main groups: (1) Traditional office-based fundus cameras have the best image quality, but also the highest cost overall, and personal clinical visits are required by the patients. The operation of such devices requires highly trained professionals because of their complexity. (2) Miniature tabletop fundus cameras are simplified, but still require personal visits in a clinical setting. High cost also limits the wider spread of these devices. (3) Point and shoot off-the-shelf cameras are light, hand-held devices. They have low cost and relatively good image quality. The main limitation of these cameras is the lack of fixation, so proper focusing is a cumbersome task. Reflections from various parts of the eye can hide important parts of the retina. (4) Integrated adapter-based hand-held ophthalmic cameras can produce a high resolution, reflection-free image. The bottleneck is the manual alignment of the light beam, which makes image acquisition highly time-consuming. (5) Smartphone based ophthalmic cameras emerged from the continuous development of the mobile phone hardware. The application of such devices may have a major impact in clinical fundus photography in the future. The main limitations of the mobile platform are rooted in its hand-held nature: focusing and illumination beam positioning can be time-consuming. However, despite that their performance is not yet assessed in comprehensive clinical trials, these devices show promising results.

### 3.2 Image pre-processing

Pre-processing is a key issue in the automated analysis of color fundus photographs. The studies of Scanlon et al. [14] and Philip et al. [15] reported that 20.8% and 11.9% of the patients, respectively, had images from at least one eye that cannot be analyzed clinically because of insufficient image quality. The major causes of poor quality are the non-uniform illumination, reduced contrast, media opacity (e.g., cataract), and movements of the eye. The application of pre-processing techniques can mitigate or even eliminate these problems, but improves the efficiency of the image analysis methods on good quality images, as well. Among several other image processing methods, Sonka et al. [16] and Koprowski [17] offer a great outlook on pre-processing methods.

The pre-processing method proposed by Youssif et al. [18] aims to reduce the vignetting effect caused by non-uniform illumination of a retinal image. Small dark objects like MAs can be enhanced by this step.

Walter et al. [19] defined a specific operator for contrast enhancement using a gray level transformation. Intensity adjustment was used to enhance the contrast of a grayscale image by saturating the lowest and highest 1% of the intensity values in [20]. The histogram equalization method proposed in [18] also aimed to enhance the global contrast of the image by redistributing its intensity values. To do so, the accumulated normalized intensity histogram was created and transformed to uniform distribution. Contrast limited adaptive histogram equalization [21] is also commonly used in medical image processing to make the interesting parts more visible. This method is based on local histogram equalization of disjoint regions. A bilinear interpolation is also applied to eliminate the boundaries between regions.

By [22], MAs appearing near vessels become more easily detectable with the removal of the complete vessel system before candidate extraction. After removing the vessel system, interpolation techniques [23] can be used. Lin et al. [24] recommended a method for vessel system detection, which considered the vasculature as the foreground of the image. The background was extracted by applying an averaging filter, followed by threshold averaging for smoothing. The background image was then subtracted from the original one.

The choice of the appropriate image pre-processing methods also depends on the subsequently used algorithms. Antal and Hajdu [25] proposed to select an optimal combination of pre-processing methods and lesion candidate extractors by stochastic search. The main role of pre-processing methods in this ensemble-based system is to increase the diversity of the lesion candidate extractor algorithms. Further, Tóth et al. [26] proposed a method to find the optimal parameter setting of such systems. More details on the ensemble-based approaches will be given in Section 3.5.

### 3.3 Localization and segmentation of the anatomic landmarks

#### 3.3.1 Localization and segmentation of the optic disc and optic cup

In general, the localization and the segmentation of the optic disc (OD, see also Fig. 1 (b)) mean the determination of the disc center and contour, respectively. These tasks are important to locate the anatomic structures in retinal images as well as in registering pathological changes within the OD region. Especially, the abnormal enlargement of the optic cup (OC) may relate to glaucoma.

The OD localization methods can be divided into two main groups: approaches that are based on the intensity and shape features of the OD and those that use the location and orientation of the vasculature.

Lalonde et al. [27] applied Haar DWT-based pyramidal decomposition to locate candidate OD regions, i.e., pixels with the highest intensity values at the lowest resolution level. Then, Hausdorff distance-based template matching was used to find circular regions with a given dimension to localize the OD. Lu and Lim [28] designed a line operator to capture bright circular structures. For each pixel, the proposed line operator evaluated the variation of the image brightness along 20 line segments of specific directions. The OD was located using the line segments with maximum and minimum variations.

Hoover and Goldbaum [29] proposed to use fuzzy convergence [30] to localize the OD center. Here, the vessel system was thinned and fuzzy segments modeled each of the line-like shapes. As a result, a voting map was generated and the pixel having the most votes was considered as the OD center. Foracchia et al. [31] exploited the directional pattern of the retinal vasculature to localize the OD. After segmenting the vasculature and determining the centerlines, diameters, and directions of the vessels, a parametric geometric model was fit to the main vessels to localize the OD center. Youssif et al. [32] proposed a method, where the OD was localized by the geometry of the vessels. After vessel segmentation, matched filtering was applied with different template sizes at various directions. Then, thinning was used to extract the centerline of each vessel.

Several other approaches considering various principles exist for the detection of the OD, like kNN location regression [33], Hough-transform [34], [35], and circular transformation [36], as well.

Yu et al. [37] identified the candidate OD regions using template matching and localized the OD based on vessel characteristics on its surface. The obtained OD center and estimated radius were used to initialize a hybrid level-set model, which combined regional and local gradient information. Cheng et al. [38] proposed superpixel classification to segment the OD and OC. After dividing the input image into superpixels, histograms and center surround statistics were calculated to classify the superpixels as OD/OC or non-OD/non-OC ones.

Hajdu et al. [39] proposed an ensemble-based system specifically designed for spatial constrained voting. Here, the output of each individual algorithm is a vote for the center of the OD. Tomán et al. [40] extended this system with assigning weights to the detector algorithms according to their individual accuracies. Hajdu et al. [41] made a further extension by introducing corresponding diversity measures to discover the dependencies of the detector algorithms better. A detailed comparison of the aforementioned algorithms is enclosed in Table 1 (see Appendix).

The cup-to-disc ratio is the ratio of the diameters of the OD and OC and the main indicator of glaucoma [42]. Glaucoma caused blindness is irreversible, but preventable with early detection and proper treatment. Furthermore, a recent study [43] showed that participants with glaucoma were more likely to develop dementia. For the determination of the cup-to-disc ratio see [44], [45], [46], while a mobile phone-based approach will be presented in detail in Section 4.

#### 3.3.2 Localization and segmentation of the macula and the fovea

The fovea, as the center of the macula, is situated at the distance two and half times of the OD diameter between the major temporal vascular arcades (see Fig. 1 (b)). Since the macula is the center of sharp vision, it has an important role in image analysis. The automatic localization of the macula/fovea is generally based on visual characteristics and positional constraints. Sinthanayothin et al. [47] localized the macula within a predefined distance from the OD as the region having maximal correlation between a template and the intensity image obtained by HSI transformation. Li and Chutatape [48] estimated the location of the macula by fitting a parabola on the main vessels having its vertex at the OD center. The macula was found on the main axis of the parabola based on its intensity and distance from the OD. Tobin et al. [49] relied solely on the segmented vessels and the position of the OD. They determined a line that was roughly passing through the OD and the macula using a parabolic model of the vasculature and localized the fovea by its distance from the OD. Chin et al. [50] localized the fovea as the region of minimum vessel density within a search region that was derived from anatomic constraints. Instead of a predefined value, the distance of the OD and macula was learned from annotated images.

Niemeijer et al. [51] used an optimization method to fit a point distribution model to the fundus image. The points of the model specified the location of the anatomic landmarks of the fundus including the fovea. In [52], the same authors presented a faster method using a kNN regressor to predict the distance of the OD and fovea at a limited number of locations in the image based on a set of features. Welfer et al. [53] considered the relative locations of the retinal structures and mathematical morphology for macula detection. After the candidate regions were identified, morphological filtering was applied to remove lesions, and the center of the darkest region was selected as the fovea. Antal and Hajdu [54] applied a stochastic search-based approach to improve macula detector algorithms with finding the optimal adjustment of parameters by simulated annealing.

Most of the macula/fovea detection approaches rely on the spatial relationship between the anatomic landmarks and their detection accuracies may drop, if the geometry considered strictly fixed. For example, the ratio of the OD diameter and the OD to fovea distance may vary depending on the age of the patient and pathologies such as optic nerve hypoplasia and physiologic macrodisc. Another important issue is that some of these methods (e.g., [49], [50]) were developed to work only with images centered at the fovea. A detailed comparison of the algorithms is enclosed in Table 2 (see Appendix).

The proper localization of the macula is important also in the recognition of age-related macular degeneration (AMD), which is the leading cause of blindness among adults and is an increasing health problem. AMD cannot be cured, but its progress can be prevented by early diagnosis and treatment. There are two major forms of the disease: non-exudative (dry) AMD that is indicated by the presence of yellowish retinal waste deposits (drusen) in the macula, and exudative (wet) AMD that is characterized by choroidal neovascularization that leads to blood and protein leakage (exudates). Non-exudative AMD is the more common form and it causes vision loss in the central region first; however, it can lead to the exudative form that can cause rapid vision loss if left untreated. Automatic image analysis methods aiming to detect the presence of this disease are currently based on support vector machine classification [55], hierarchical image decomposition [56], [57], statistical segmentation methods [58], deep learning [59], and pixel intensity characteristics [60].

#### 3.3.3 Segmentation of the vessel system

##### 3.3.3.1 Segmentation

In general, most vessel segmentation methods consider the green channel of the image, because the contrast is higher and the noise level is lower here.

Soares et al. [61] proposed a method that classified pixels as vessel or non-vessel ones using supervised classification. Here, Gabor transform was applied for contrast enhancement. Lupaşcu et al. [62] used AdaBoost to construct a classifier. In this method, 41 features were extracted based on local spatial properties, intensity structures and geometry.

**Fig. 3:**
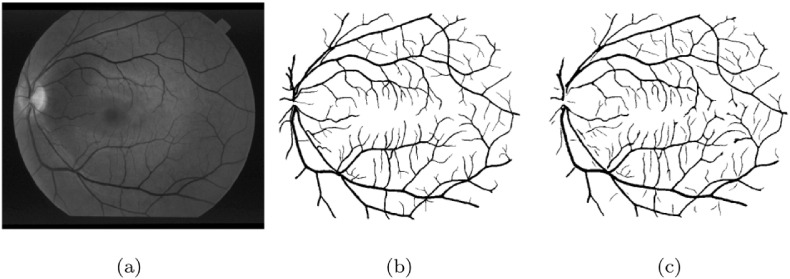
Segmentation of the vascular system by [64]; (a) original image, (b) manually annotated vascular system, (c) automatic segmentation result.

Methods based on matched filtering convolve the retinal image with 2D templates, which are designed to model the characteristics of the vasculature. The presence of a feature at a given position and orientation is indicated by the filter response. Chaudhuri et al. [63] had one of the earliest approaches for the automated segmentation of the vascular system. They proposed a template with a Gaussian profile to detect piecewise linear segments of vessels. The filter response image was thresholded and post-processed to obtain the final segmentation. Kovács and Hajdu [64] also proposed a method based on template matching and contour reconstruction. The centerlines of the vessels were extracted by generalized Gabor templates followed by the reconstruction of the vessel contours. The intensity characteristics of the contours that were learned in training databases with a typical output is shown in Fig. 3.

As lesions can exhibit similar local features as vessels, their occurrence might deteriorate the quality of segmentation. Annunziata et al. [65] proposed a method to address the presence of exudates. After pre-processing, exudates are extracted and inpainted and a multi-scale Hessian eigenvalue analysis was applied to enhance vessels. A detailed comparison of the algorithms can be found in Table 3 (see Appendix).

##### 3.3.3.2 Artery and vein classification

**Fig. 4:**
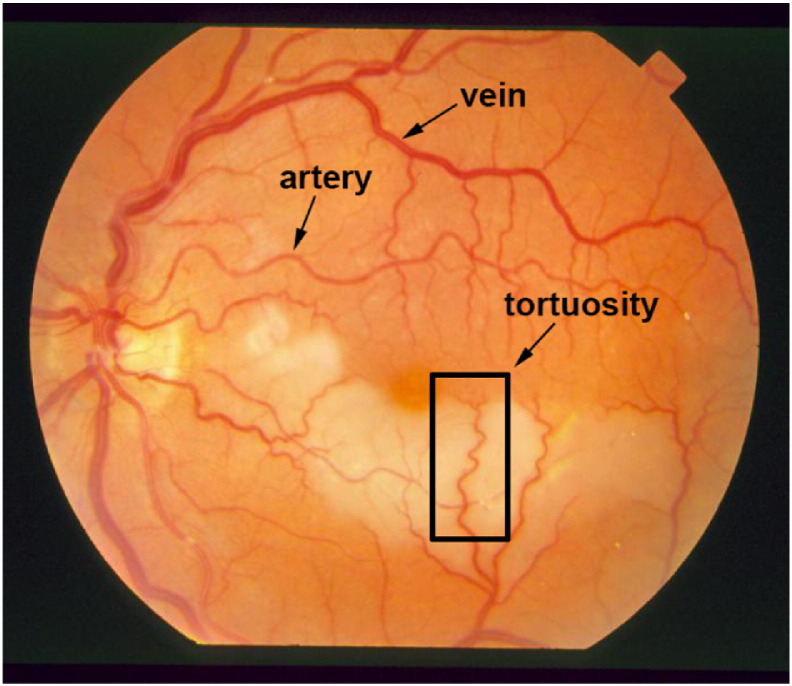
A retinal image from the STARE database [71] illustrating severe vessel tortuosity.

By the classification of the vessels, important diagnostic indicators can be obtained, such as the arteriolar-to-venular diameter ratio. In general, vessels show different characteristics, size and color; arteries are brighter and usually thinner as it can also be observed in Fig. 4. Zamperini et al. [66] classified vessels based on color, position, size and contrast by investigating the surrounding background pixels. Relan et al. [67] used Gaussian Mixture Model – Expectation Maximization clustering to classify vessels. Dashtbozorg et al. [68] proposed a classification method based on the geometry of vessels. First, a graph was assigned to the vessel tree around the OD. Next, different intersection points were determined: bifurcation, crossing, meeting, and connecting points. Finally, classification was performed based on a list of features, like node degree, vessel caliber and orientation of links. Estrada et al. [69] also considered a graph theoretical approach extended by a global likelihood model. Relan et al. [70] applied a Least Square – Support Vector Machine technique to classify veins based on four color features. Table 4 contains a detailed comparison of the algorithms (see Appendix).

##### 3.3.3.3 Vasculature measurements

The measurement of vascular tortuosity (see Fig. 4) is important in the detection of hypertension, diabetes and some central nervous system diseases. Some of the earliest works were summarized by Hart et al. [72] with proposing a tortuosity measure to classify vessel segments and networks, as well. Since then, several different approaches have been proposed and currently tortuosity measurement algorithms can be categorized in five main groups: (1) arc length over chord length ratio, (2) measures involving curvature, (3) angle variation, (4) absolute direction angle change, (5) measures based on inflection count. Grisan et al. [73] highlighted some methods from each group. Moreover, they proposed a tortuosity density measure to handle vessel segments of different lengths with summing every local turn. Lotmar et al. [74] introduced the first method of the first category, which was later widely utilized. Poletti et al. [75] proposed a combination of methods for image-level tortuosity estimation. Oloumi et al. [76] considered angle variation for tortuosity assessment in the detection of retinopathy of prematurity. Lisowska et al. [77] compared five methods settling on different principles. Perez-Rovira et al. [78] proposed a complete system for vessel assessment that used the tortuosity measure by Trucco et al. [79]. Aghamohamadian-Sharbaf et al. [80] created a curvature-based algorithm applying a template disc method. They also showed that curvature had a non-linear relation with tortuosity. A detailed comparison of the algorithms is enclosed in Table 5 (see Appendix).

Vessel bifurcations are important in the detection of certain central nervous system diseases. Tsai et al. [81] proposed a method for vessel bifurcation estimation consisting of three components: a circular exclusion region to model the intersections, a landmark location for the intersection itself, and orientation vectors to represent the vessels meeting at the intersection. This algorithm iteratively mapped vessels in order to obtain bifurcations and crossings. Several other vasculature measurements have been reported, like fractal dimension of the vasculature for the detection of DR [82] or for the detection of stroke [83], vessel diameter [84], and arteriolar-to-venular diameter ratio [85], [86]. Xu et al. [87] proposed a graph-based segmentation method to measure the width of vessels.

### 3.4 Detection of retinal lesions

#### 3.4.1 Detection of microaneurysms

MAs (see Fig. 1 (b)) are swellings of the capillaries and appear as dark red isolated dots. They are very common lesions of various diseases, thus, reasonable efforts have been made for their proper detection considering several principles.

Walter et al. [88] introduced an algorithm for MA candidate extraction. It starts with image enhancement and green channel normalization, followed by candidate detection with diameter closing and an automatic thresholding scheme. Finally, the classification of the candidates was performed based on kernel density estimation. Among the most widely applied candidate extractor methods we find Spencer et al. [89] and Frame et al. [90]. Here, shade correction was applied by subtracting a median filtered background from the green channel image. Candidate extraction was accomplished by morphological top-hat transformations using twelve structuring elements. Finally, a contrast enhancement operator was applied followed by the binarization of the resulting image. Slightly different approaches can be found in [91], [92], [93].

**Fig. 5:**
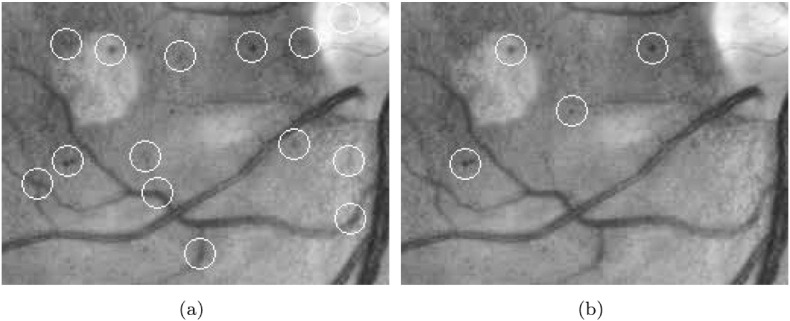
Results of microaneurysm candidate extraction; (a) by [88], (b) by [96].

Abdelazeem et al. [94] recommended the usage of circular Hough transformation [95] to extract MAs as disc-shaped spots. Lázár and Hajdu [96] proposed a method using pixel intensity profiles. After smoothing the green channel with a Gaussian filter, the image was analyzed along lines at several directions. Based on intensity peaks, adaptive thresholding was applied to binarize the image and the final components were filtered based on their sizes. Zhang et al. [97] considered multi-scale correlation filtering and dynamic thresholding. Five Gaussian masks with different variances were applied and their maximal responses were thresholded to extract MA candidates. The results of two different candidate extractors are also shown in Fig. 5. Table 6 contains a detailed comparison of the algorithms (see Appendix).

As a recent multi-modal approach, Török et al. [98] combined MA detection with tear fluid proteomical analysis [99] for DR screening.

#### 3.4.2 Detection of exudates

Generally, exudate detection techniques can be separated in two groups. Algorithms in the first group are based on mathematical morphology, while those in the second on pixel classification.

Walter et al. [100] proposed a method that used morphological closing as a first step to eliminate vessels. Then, local standard deviation was calculated to extract the candidates. Finally, a morphological reconstruction method was used to find exudate contours. Since the OD also appears as a bright spot, Sopharak et al. [101] eliminated the OD as a first step. Then, Otsu thresholding was used to locate high intensity regions. After contrast enhancement, Welfer et al. [102] applied an H-maxima transform on the L channel in the color space CIE 1976 L*u*v*.

**Fig. 6:**
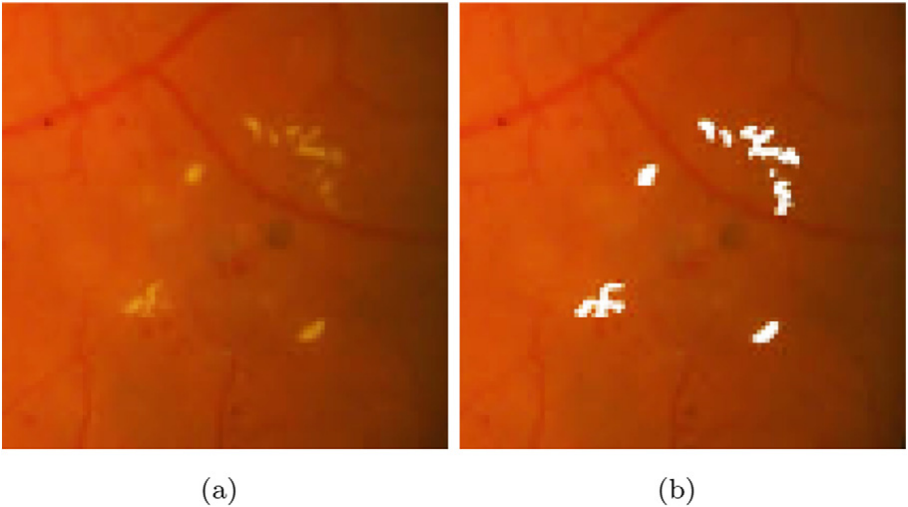
Exudate detection by [109] after contrast enhancement and cropping; (a) original fundus image, (b) the result of detection.

In order to determine whether a pixel is in the exudate region or not, Sopharak et al. [103] introduced a method using fuzzy c-means clustering. Then, morphological operations were applied to refine the results. In [104], Sopharak et al. showed that Naive Bayes classification can also be applied for this task. Sánchez et al. [105] detected small isolated exudates and used them for training. Therefore, a new training set was defined for classification for each image. Niemeijer et al. [106] recommended a multi-level classification method, where candidates were labeled as drusen, exudates or cotton wool spots. García et al. [107] used neural networks to identify exudates. Harangi et al. [108], [109] proposed a system for exudate detection using a fusion of active contour methods and region-wise classifiers; for some detection results see Fig. 6.

In addition to the aforementioned methods, Ravishankar et al. [22] suggested the detection of lesions including exudates within a complex landmark extraction system for DR screening. A detailed comparison of the algorithms is enclosed in Table 7 (see Appendix).

#### 3.4.3 Detection of other lesions

Cotton wool spots are reminiscent in appearance of exudates; therefore, similar approaches can be considered for their detection. However, for the same reason, the differentiation of cotton wool spots and exudates is a challenging task [106]. Hemorrhages are dark lesions, but their varied shape and size are similar to that of exudates. For example, after some appropriate modifications the exudate detection method [108] could be applied for the segmentation of hemorrhages, as well. A survey on recent methods for hemorrhage detection can be found in [110].

### 3.5 Ensemble-based detection

Though single methods can perform well in general, there are challenging situations when they fail. In fact, there is no reason to assume that an individual algorithm could be optimal for such heterogeneous data as retinal images.

To address this issue, a possible approach is to apply ensemble-based systems, which principle had a strong focus in our contributions presented in this section. An ensemble-based system consists of a set of algorithms (members), whose individual outputs are fused by some consensus scheme, e.g. by majority voting. An ensemble composed of algorithms based on sufficiently diverse principles is expected to be more accurate than any of its members if they perform better than random guessing [111]. The diversity of the members allows an ensemble to respond more flexibly to various conditions originating from e.g., the presence of specific diseases in a dataset.

**Fig. 7:**
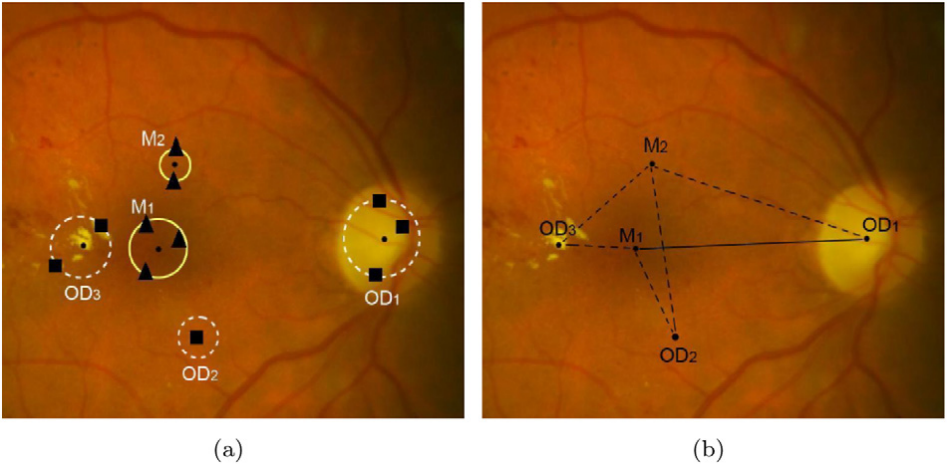
Simultaneous ensemble-based detection of the OD and macula by [112]; (a) candidate regions voted by various detector algorithms, (b) final candidates using geometric relationships (distance and angle).

For example, the detection of the OD may be based on its main characteristic being a bright oval patch. However, if bright lesions like exudates are also present, the objects might be misclassified. To overcome these problems, we can create ensembles of algorithms to fuse their findings. Qureshi et al. [112] proposed a combination of algorithms for the detection of the OD and macula. The selection of the algorithms was based on detection accuracy and computation time. Moreover, a weight value was assigned to each algorithm according to its candidate extraction performance. The final locations of the OD and macula were determined by a weighted graph theoretical approach, which took the mutual geometric placements also into consideration (see Fig. 7). Harangi and Hajdu [113] introduced an ensemble-based system also for OD detection, but extracted more candidates for each member algorithm. Weights were assigned to the candidates according to the ranking and accuracy of their extractor algorithms.

**Fig. 8:**
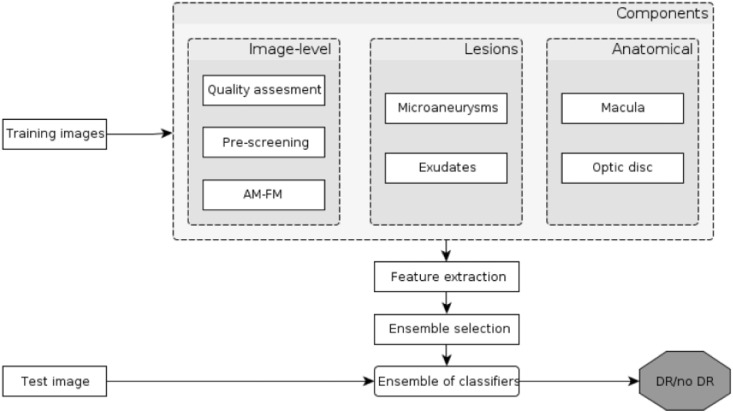
Flowchart of the ensemble-based system for retinal image analysis from [117].

Ensemble-based systems have been applied for lesion detection, as well. Nagy et al. [114] proposed a system for exudates that was an optimal combination of pre-processing methods and candidate extractors. The ensemble pool consisted of several (pre-processing method, candidate extractor) pairs in all possible combinations. To find the best ensemble, a simulated annealing-based search algorithm was used. Next, a voting scheme was applied with the following rule:if more than 50% of the ensemble member pairs marked a pixel as an exudate one, their labeling was accepted. Antal and Hajdu [115], [116] applied roughly the same approach for MA detection. Further, Antal and Hajdu [117] proposed a complete system for DR-screening, where fusion-based approaches were considered for both the detections of anatomic parts/lesions and to make the final decision for an image based on the output of different classifiers as illustrated in Fig. 8. On the basis of these works, we can conclude that ensemble-based methods often outperform individual algorithms, especially in more challenging situations. This claim is also supported by the corresponding performance measures in Table 1, Table 6, and 7 (see Appendix).

### 3.6 Performance evaluation of algorithms

#### 3.6.1 Databases for performance evaluations

In this section, we list several publicly available databases that are generally used to quantitatively compare the performances of the algorithms collected in this review.

Retinopathy Online Challenge (ROC) [118] is a worldwide online competition dedicated to measure the accuracy of MA detectors. The ROC database consists of 50 training and 50 test images having different resolutions (768 × 576, 1058 × 1061 and 1389 × 1383 pixels), 45° field-of-view (FOV) and JPEG compression. For objective comparisons of the MA detector algorithms, a test set is provided, where the MAs are not given.

The DIARETDB0 database [119] contains 130 losslessly compressed color fundus images with a resolution of 1500 × 1152 pixels and 50° FOV. 110 images contain abnormalities, like hard exudates, soft exudates, MAs, hemorrhages and neovascularization. For every fundus image, a corresponding ground truth file is available containing the OD/macula centers and all lesions appearing in the specific retinal image.

The DIARETDB1 v2.1 database [120] contains 28 losslessly compressed training and 61 test images, respectively, with a resolution of 1500 × 1152 pixels and 50° FOV. As ground truth, an expert in ophthalmology marked the OD/macula centers and the regions related to MAs, hemorrhages, and hard/soft exudates.

The HEI-MED database [121] consists of 169 images of resolution 2196 × 1958 pixels with a 45° FOV, among which 54 images are classified manually by an ophthalmologist as containing exudates.

The Messidor database [122] consists of 1200 losslessly compressed 24-bit RGB images with 45° FOV at different resolutions of 1440 × 960, 2240 × 1488, and 2304 × 1536 pixels. For each image, a grading score is provided regarding the stage of retinopathy based on the presence of MAs, hemorrhages and neovascularization.

The DRIVE [123] database contains 40 JPEG-compressed color fundus images of resolution 768 × 584 pixels and 45° FOV. For training purposes, a single manual segmentation of the vessel system is available for each image. For the test cases, two manual segmentations are available; one is used as ground truth, the other one is to compare computer-generated segmentations with those of an independent human observer.

The STARE database [71] consists of 397 fundus images of size 700 × 605 pixels. STARE contains annotations of 39 possible retinal distortions for each image. Furthermore, the database includes manual vessel segmentations for 40 images, and artery/vein labeling for 10 images created by two experts. Ground truth for OD detection is provided for 80 images, as well.

The HRF database [124] contains high-resolution fundus images for vessel segmentation purposes. It consists of 45 JPEG-compressed RGB images of size 3504 × 2336 pixels and the images are divided to three sets of equal sizes, namely, healthy, glaucomatous and DR ones. This database contains the manual annotations of one human observer.

#### 3.6.2 Performance measurement

As the primary aim of the automatic retinal image analysis methods is to support clinical decision-making, it has key importance to objectively measure their performances, i.e., the level of agreement between their outputs and a reference standard (ground truth), which is typically a set of manual annotations created by expert ophthalmologists.

The most commonly used measures to assess the performance of retinal image segmentation methods are sensitivity, specificity, precision, accuracy, and the F1-score. These measures rely on the number of true positive (*TP*, correctly identified), false positive (*FP*, incorrectly identified), true negative (*TN*, correctly rejected), and false negative (*FN*, incorrectly rejected) hits. The sensitivity and specificity of a method are calculated as *TP*/(*TP* + *FN*) and *TN*/(*TN* + *FP*), respectively, while precision is as *TP*/(*TP* + *FP*). Accuracy is determined as (*TP* + *TN*)/(*TP* + *FP* + *TN* + *FN*), while the F1-score measures the performance of a method by equally weighting sensitivity and precision via 2*TP*/(2*TP* + *FP* + *FN*).

When a method also assigns confidence values to its output, its specificity and sensitivity can be adjusted by thresholding these confidence values. Plotting the resulting sensitivity against 1−specificity as the threshold is changed yields a receiver operator characteristics curve. As sensitivity and specificity fall between 0 and 1, the receiver operator characteristics curve resides within the unit square. The area under the receiver operator characteristics curve (AUC) quantifies the overall performance of a given method:an AUC value of 1 means perfect performance, while 0.5 indicates random behavior. All these measures are routinely applied to the evaluation of the different types of algorithms described in this review.

As the different image analysis methods are evaluated using various (often non-public) dataset, their performance measures are not directly comparable in general. For this reason, it is also not easy to select a single best method for a given task based on solely its reported performance measures. For example, it is often uncertain how the sensitivity and specificity of a method would change depending on the ratio of diseased and non-diseased images in the dataset. Therefore, we recommend the evaluation of methods on a subset of images representing the desired data to be processed in order to select the appropriate image analysis methods. However, in this selection Tables 1–7 may give some clues by showing certain accuracy figures for both diseased and non-diseased datasets.

It is also worth noting how the retinal image analysis performances of the currently available automated diagnostic systems compare to that of human experts. Abràmoff et al. [125] presented a DR screening system having nearly the same performance as human experts in terms of sensitivity and specificity, achieving an AUC value 0.850. Other state-of-the-art approaches Hansen et al. [126] and Agurto et al. [127] reported AUC figures 0.878 and 0.890, respectively. The ensemble-based DR screening system described by Antal and Hajdu [117] provided an AUC value 0.900 in a disease/no disease setting. However, these AUC figures were found on datasets having different proportions of patients showing/missing signs of DR.

## 4 Future trends in retinal image analysis

Considering the recent advances in the discovery of retinal biomarkers and biomarker candidates, more widespread adoption of retinal imaging can be expected in the clinical practice in the future for the early identification of several chronic diseases and long-term conditions. With the increasing amount of retinal images, the application of automatic image analysis techniques are expected to become more important to aid the work of the medical experts and to decrease the associated care costs. The automatic analyses of retinal images may also facilitate the establishment of large-scale computer aided screening and prevention programs. In this respect, telemedicine and mobile devices may play a critical role in the future, e.g., by allowing patients to send retinal images for regular control without the need of visiting a screening center.

**Fig. 9:**
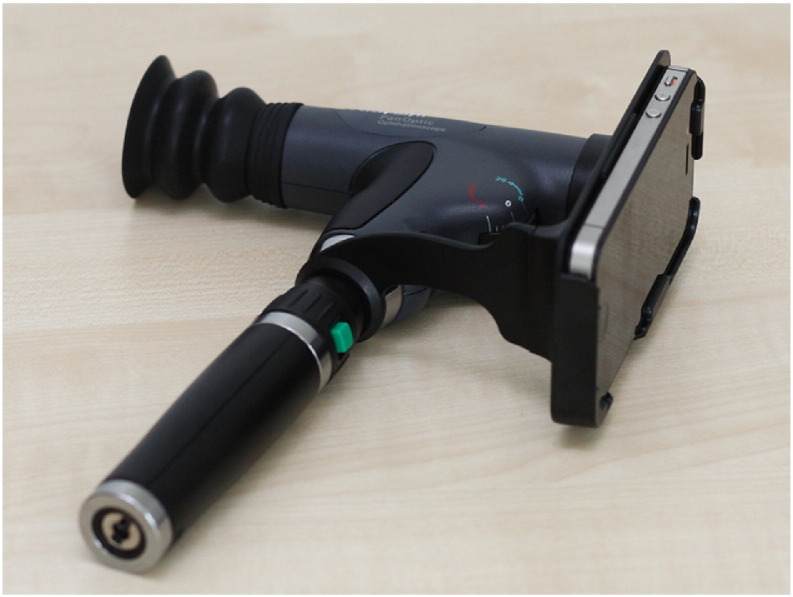
A retinal camera attached to a mobile phone.

**Fig. 10:**
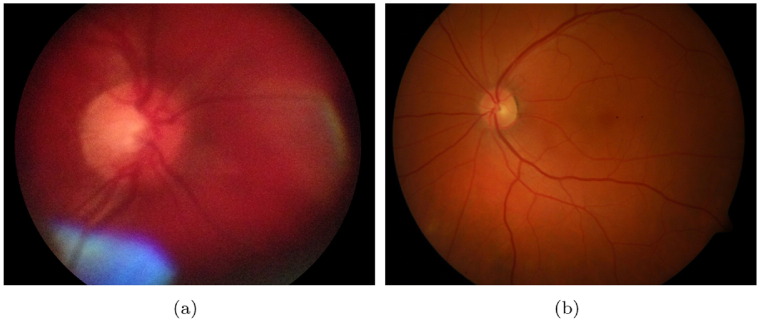
Sample fundus images acquired by (a) a mobile fundus camera (FOV 25°), (b) a clinical device (FOV 50°).

In the recent years, mobile devices have a rapid and extensive development. Their hardware resources and processing power give us the chance to consider them as possible tools for ophthalmic imaging. Bolster et al. [128] reviewed the recent advancements in smartphone ophthalmology. In most solutions, extra hardware is necessary to acquire good quality images. One such tool is the Welch Allyn iExaminer System shown in Fig. 9, which can be attached to an Apple iPhone 4/4S. To date, this is the only FDA-approved ophthalmoscope for mobile phones [129]. In general, compared to professional fundus cameras, smartphone-based ophthalmoscopes have a narrower FOV, lower contrast, and less brightness/sharpness in comparison with a clinical device (see Fig. 10).

Haddock et al. [130] described a technique, which lets high-quality fundus images be taken. This is a relatively cheap solution with consisting of a smartphone (iPhone 4 or 5), a 20D and an optional Koeppe lens. Prasanna et al. [131] outlined a concept of a smartphone-based decision support system for DR screening. Giardini et al. [132] proposed a complex system based on an inexpensive ophthalmoscope adapter and mobile phone software.

**Fig. 11:**
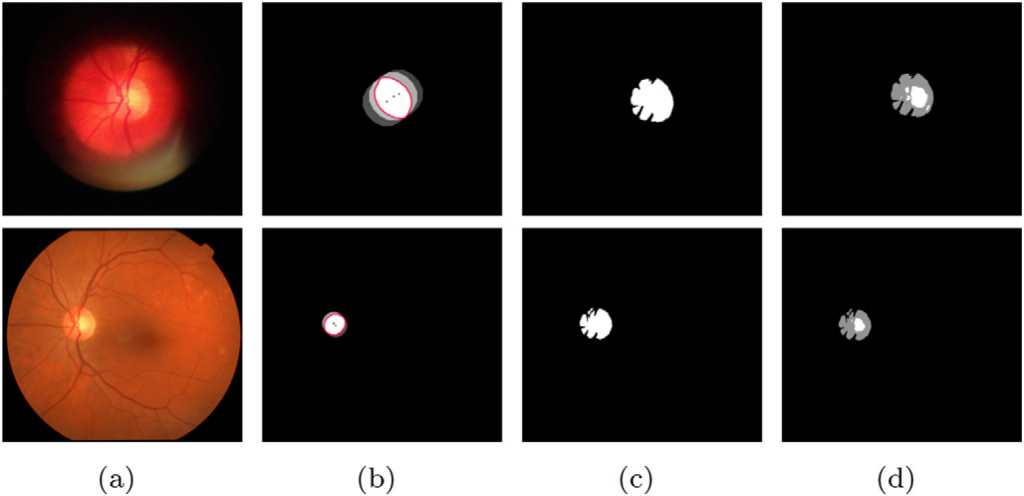
The results of [133] for OD and OC segmentation on a mobile (top row) and a clinical (bottom row) fundus image; (a) original images, (b) OD centers and average size OD discs, (c) precise OD boundary extracted by active contour, (d) OD and OC pixels after classification.

Besenczi et al. [133] recommended an image processing method for cup-to-disc ratio measurement on images taken by mobile phones. An important motivation of the study was the comparison of the mobile platform with the clinical setting, so images were acquired from the same patients by both mobile and office-based cameras. Cup-to-disc ratio calculation was based on the fusion of several OD detectors. After the segmentation of the OD region, each pixel was classified based on its intensity as an OD, OC or vessel one. The steps of the proposed method are also shown in Fig. 11. It has been found that the accuracy drops only moderately on the mobile platform comparing with the clinical one.

## 5 Conclusions

The efficiency of the state-of-the-art methods summarized in this paper are measured on images belonging to public and non-public datasets. Although the objectivity of these quantitative measures are evident, less is known about how these algorithms are expected to perform in general. For example, most papers do not mention how the selected image acquisition technique or image resolution affects the overall performance of these methods. Thus, it would be a very precious future practice to evaluate regarding several factors to allow other researchers to fine tune the parameter settings of the algorithms for their specific image data, as it is done e.g. in [134] for noise filtering. Though in this work we focus on fundus photography, from other image acquisition techniques we can highlight optical coherence tomography with the corresponding image analytic methods [135], [136].

As for performance, the accuracies of the algorithms are generally considered as primarily important. On the other hand, some approaches, like the fusion-based ones discussed in the paper, can be expected to raise accuracy at the expense of computational time. Unfortunately, proper benchmarking analyses are often omitted in the presentation of the algorithms, and the rapid development of computer hardware and the various hardware platforms also make a quantitative comparison of the execution times challenging. However, observing the methodologies the algorithms are generally based on we can draw some conclusions. For example, the growing amount of clinically annotated images should lead to the raise of detection accuracy for algorithms considering machine learning without increasing the processing time of an image to be evaluated. On the other hand, the offline learning process may become computationally very demanding. Algorithms considering filters based on local neighborhoods can improve their accuracies with reacting to higher resolution with simply increasing the size of the filters for the cost of execution time. As a critical issue regarding computational performance, possibilities of distributed processing should be checked in each method. Parallel implementations can be easily provided for pixel- and region-level feature extraction or image-level processing. For algorithms having free parameters, the optimal settings of them for different datasets can be determined by stochastic optimization, which approaches also offer heuristic parallel search strategies at the expense of a slight risk for dropping some accuracy. In several methods, an efficient solution to reduce the computational time is to substitute processes operating in the spatial domain with alternatives in the frequency domain. Algorithms interpreting an image in a wider biological context are challenging to make computationally efficient. For example, if the detected components and relations are processed by graph algorithms, the solutions can be found only in heuristic ways.

As a brief summary of this review we can claim that the comprehensive predictive and exploratory investigation of medical data – including the automatic analysis of retinal images – has the potential to effectively support clinical decision-making and with the progress towards personalized medicine it will become indispensable.

## Figures and Tables

**Table 1:** Algorithms for the localization and segmentation of the OD.

Authors	Method	Database(s) used	No. of images	Performance measure
Lalonde et al. [27]	Pyramidal decomposition, template matching	Non-public dataset	40	ACC 1.00
Lu and Lim [28]	Line operator	DIARETDB0, DIARETDB1, DRIVE, STARE	340	ACC 0.9735
Hoover and Goldbaum [29]	Fuzzy convergence of the retinal vessels	STARE	81	ACC 0.89
Foracchia et al. [31]	Modeling the direction of the retinal vessels	STARE	81	ACC 0.9753
Youssif et al. [32]	2D Gaussian matched filtering, morphological operations	DRIVE; STARE	121	ACC 1.00; ACC 0.9877
Abràmoff and Niemeijer [33]	kNN location regression	Non-public dataset	1000	ACC 0.9990
Sekhar et al. [34]	Morphological operations, Hough transform	DRIVE; STARE	55	ACC 0.947; ACC 0.823
Zhu and Rangayyan [35]	Edge detection, Hough transform	DRIVE	40	ACC 0.9250
Lu [36]	Circular transformation	ARIA, Messidor, STARE	1401	ACC 0.9950
Qureshi et al. [112]	Majority voting-based ensemble	DIARETDB1; DIARETDB1; DRIVE	259	ACC 0.9679; ACC 0.9402; ACC 1.00
Harangi and Hajdu [113]	Weighted majority voting-based ensemble	DIARETDB0; DIARETDB1	219	PPV 0.9846; PPV 0.9887
Hajdu et al. [39]	Spatially constrained majority voting-based ensemble	Non-public dataset; Messidor	1527	ACC 0.921; ACC 0.981
Tomán et al. [40]	Spatially constrained weighted majority voting-based ensemble	Messidor	1200	ACC 0.98
Yu et al. [37]	Template matching, hybrid level-set model	Messidor	1200	ACC 0.9908
Cheng et al. [38]	Superpixel classification	Non-public dataset	650	ACC 0.915

**Table 2:** Algorithms for the localization and segmentation of the macula and the fovea.

Authors	Method	Database(s) used	No. of images	Performance measure
Sinthanayothin et al. [47]	Template matching, positional constraints	Non-public dataset	112	SE 0.804, SP 0.991
Li and Chutatape [48]	Pixel intensity, positional constraints	Non-public dataset	89	ACC 1.00
Tobin et al. [49]	Parabolic model	Non-public dataset	345	ACC 0.925
Chin et al. [50]	Minimum vessel density, positional constraints	Non-public dataset; Messidor	419	ACC 0.8534; ACC 0.7294
Niemeijer et al. [51]	Point distribution model	Non-public datasets	500; 100	ACC 0.944; ACC 0.920
Niemeijer et al. [52]	kNN regression	Non-public datasets	500; 100	ACC 0.968; ACC 0.890
Welfer et al. [53]	Mathematical morphology	DIARETDB1; DRIVE	126	ACC 0.9213; ACC 1.00
Antal and Hajdu [54]	Intensity thresholding	DIARETDB0; DIARETDB1; DRIVE	199	ACC 0.86; ACC 0.92; ACC 0.68

**Table 3:** Algorithms for the segmentation of the retinal blood vessel system.

Authors	Method	Database(s) used	No. of images	Performance measure
Soares et al. [61]	2D Gabor wavelet, Bayesian classification	DRIVE; STARE	60	AUC 0.9614; AUC 0.9671
Lupaşcu et al. [62]	AdaBoost-based classification	DRIVE	20	AUC 0.9561, ACC 0.9597
Chaudhuri et al. [63]	2D matched filters	non-public dataset	NA	NA
Kovács and Hajdu [64]	Template matching, contour reconstruction	DRIVE; STARE; HRF	105	ACC 0.9494; ACC 0.9610; ACC 0.9678
Annunziata et al. [65]	Hessian eigenvalue analysis, exudate inpainting	STARE; HRF	65	ACC 0.9562; ACC 0.9581

**Table 4:** Algorithms for the classification of arteries and veins.

Authors	Method	Database(s) used	No. of images	Performance (ACC)
Zamperini et al. [66]	Supervised classifiers	Non-public dataset	42	0.9313
Relan et al. [67]	GMM-EM clustering	Non-public dataset	35	0.92
Dashtbozorg et al. [68]	Graph-based classification	DRIVE; INSPIRE-AVR [86]; VICAVR [137]	138	0.874; 0.883; 0.898
Estrada et al. [69]	Graph-based framework, global likelihood model	Non-public dataset; 1:2:DRIVE; INSPIRE-AVR	110	0.910; 1:0.935, 2:0.917; 0.909
Relan et al. [70]	LS-SVM classification	Non-public dataset; DRIVE	90	0.9488; 0.894

**Table 5:** Algorithms for the assessment of vessel tortuosity.

Authors	Method	Database(s) used	No. of images	Performance (SCC)
Grisan et al. [73]	Inflection-based measurement	RET-TORT [73]	60	0.949 (artery), 0.853 (vein)
Poletti et al. [75]	Combination of measures	Non-public dataset	20	0.95
Oloumi et al. [76]	Angle-variation-based measurement	Non-public dataset	7	NA
Trucco et el. [79]	Curvature and vessel width-based measurement	DRIVE	20	NA
Aghamohamadian-Sharbaf et al. [80]	Curvature-based measurement	RET-TORT	60	0.94

**Table 6:** Algorithms for the detection of MAs.

Authors	Method	Database(s) used	No. of images	Performance measure
Walter et al. [88]	Gaussian filtering, top-hat transformation	Non-public dataset	94	SE 0.885 (FPI 2.13)
Spencer et al. [89]	Morphological operators, matched filtering	Non-public dataset	NA	SE 0.824, SP 0.856
Niemeijer et al. [91]	kNN pixel classification	Non-public dataset	140	SE 1.00, SP 0.87
Mizutani et al. [92]	Double-ring filter, neural network classification	ROC	50	SE 0.648 (FPI 27.04)
Fleming et al. [93]	Contrast normalization, watershed region growing	Non-public dataset	1441	SE 0.854, SP 0.831
Abdelazeem [94]	Circular Hough transformation	Non-public dataset	3	NA
Lázár and Hajdu [96]	Directional cross-section profiles	Non-public dataset; ROC	110	RS 0.233; RS 0.423
Zhang et al. [97]	Multi-scale correlation coefficients	ROC	50	RS 0.357
Antal and Hajdu [115]	Ensemble-based detection	ROC	50	RS 0.434

**Table 7:** Algorithms for the detection of exudates.

Authors	Method	Database(s) used	No. of images	Performance measure
Ravishankar et al. [22]	Mathematical morphology	Non-public dataset, DIARETDB0, DRIVE, STARE	516	SE 0.957, SP 0.942
Walter et al. [100]	Mathematical morphology	Non-public dataset	30	SE 0.928, PPV 0.924
Sopharak et al. [101]	Optimally adjusted morphological operators	Non-public dataset	60	SE 0.80, SP 0.995
Welfer et al. [102]	Mathematical morphology	DIARETDB1	89	SE 0.7048, SP 0.9884
Sopharak et al. [103]	Fuzzy c-means clustering, morphological operators	Non-public dataset	40	SE 0.8728, SP 0.9924
Sopharak et al. [104]	Naive Bayes and SVM classification	Non-pubic dataset	39	SE 0.9228, SP 0.9852
Sánchez et al. [105]	Linear discriminant classification	Non-public dataset	58	SE 0.88 (FPI 4.83)
Niemeijer et al. [106]	kNN and linear discriminant classification	Non-public dataset	300	SE 0.95, SP 0.86
García et al. [107]	1:MLP, 2:RBF, and 3:SVM classification	Non-public dataset	67	1:SE 0.8814, PPV 0.8072; 2:SE 0.8849, PPV 0.7741; 3:SE 0.8761, PPV 0.8351
Harangi et al. [108]	Active contour fusion, region-wise classification	1:DIARETDB1; 2:HEI-MED	258	1:SE 0.86, PPV 0.84 (lesion level); 1:SE 0.92, SP 0.68 (image level); 2:SE 0.87, SP 0.86 (image level)
Nagy et al. [114]	Majority voting-based ensemble	DIARETDB1	89	SE 0.72, PPV 0.77
